# Crystal structure of a methyl benzoate quadruple-bonded dimolybdenum complex

**DOI:** 10.1107/S2056989023001457

**Published:** 2023-02-28

**Authors:** Lillian Dawson Bass, Jessie H. Lee, McKenzie C. Lilygren, Alaina C. Hartnett, Brandon M. Campbell, Daniel R. Morphet, Dilek K. Dogutan, Shao-Liang Zheng

**Affiliations:** a Department of Chemistry and Chemical Biology, Harvard University, Cambridge, MA, 02138, USA; Universidade de Sâo Paulo, Brazil

**Keywords:** crystal structure, quadruple bond, molybdenum, delta bond

## Abstract

Quadruple-bond dimolybdenum complexes provide invaluable insight into the two-electron bond, with structural chemistry providing a foundation for examination of bond properties. The synthesis and solid-state structure of the quadruple-bonded dimolybdenum(II) complex tetra­kis­(*μ*-4-methyl­benzoato-*κ* ^2^ *O*:*O*′)bis[(tetra­hydro­furan-κ*O*)molybdenum(II)] tetra­hydro­furan disolvate, [Mo_2_(C_8_H_7_O_2_)_4_(C_4_H_8_O)_2_]·2C_4_H_8_O, are presented. This complex crystallizes in a triclinic cell with low-symmetry space group *P*  . The dimolybdenum paddlewheel structure comprises four methyl­benzoate ligands and two axial THF ligands. The dimolybdenum bond distance of 2.1012 (4) Å is exemplary of this class of compounds.

## Abstract 2

The quadruple-bond complex, [Mo_2_(*p*-O_2_CC_6_H_4_CH_3_)_4_·2(C_4_H_8_O)]·2C_4_H_8_O, crystallizes within a triclinic space group *P* 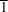 . The slightly electron donating group on the paddlewheel carboxyl­ate together with the axial THF negligibly perturbs the Mo—Mo bond distance.

## 1. Chemical context

The two-electron bond (Lewis, 1916 ▸) is the most basic element in the field of chemistry. Quadruple-bond complexes have been central in experimentally defining the two-electron bond within a unified context of the valence (Heitler & London, 1927 ▸) and mol­ecular orbital (Pauling, 1928 ▸; Lennard-Jones, 1929 ▸; Mulliken, 1932 ▸; James & Coolidge, 1933 ▸; Coulson & Fischer, 1949 ▸) bonding models. The importance of quadruple-bond complexes in elucidating the two-electron bond arises from the four states that originate from the two-orbital electron configuration: ^1^φφ, ^3^φφ*, ^1^φφ*, and ^1^φ*φ*, where φ and φ* represent bonding and anti­bonding orbitals, respectively. In experimental systems with σ and π bonding frameworks, the excited states are not all accessible because of the dissociation or rotation arising from population of σ and π anti­bonding orbitals. Quadruple-bonded metal–metal complexes, whose metal–metal linkages are characterized by a σ^2^π^4^δ^2^ ground state, are able to overcome this limitation. Pioneered by a σ^2^π^4^ framework and locked from rotation by diametrically opposed bulky ligands or bidentate ligands, all four states defining the δ^2^ two-electron bond (^1^δδ, ^3^δδ*, ^1^δδ*, and ^1^δ*δ*) may experimentally be verified for dimolybdenum quadruple-bond complexes (Engebretson *et al.*, 1994 ▸, 1999 ▸; Cotton & Nocera, 2000 ▸; Boettcher *et al.*, 2022 ▸).

In the preliminary investigation of Mo_2_(O_2_CCH_3_)_4_, Lawton & Mason (1965 ▸) determined the dimolybdenum bond distance to be 2.11 Å, which was later adjusted by Cotton & Norman (1971 ▸) to 2.0934 Å. As a result of the weak overlap of the *d_xy_* orbitals constituting a δ bond, one-electron oxidation or reduction of a dimolybdenum core does little to perturb the dimolybdenum bond distance, allowing for the spectroelectrochemical determination of the degree of overlap between these orbitals (Boettcher *et al.*, 2022 ▸).

How the properties of the equatorial ligands affect the dimolybdenum bond distance has been a central question in the structural chemistry of quadruple-bond complexes (Han, 2011 ▸). Cotton proposed that either electron-withdrawing or electron-donating substituents in the ligand field of the dimolybdenum core will modulate the bonding within the quadruple-bond framework (Cotton *et al.*, 1978 ▸). A comparative analysis of electron-donating, -neutral and -withdrawing ligands drives to the heart of this issue. Previous studies have examined the electron-neutral Mo_2_(*p*-O_2_CC_6_H_5_)_4_ (Cotton *et al.*, 1978 ▸) and electron-withdrawing Mo_2_(*p*-O_2_CC_6_H_4_CF_3_)_4_ (Aigeldinger *et al.*, 2022 ▸) groups on the paddlewheel motif to understand the electronic effect of homologous *R* groups on Mo—Mo bond distances. With this motivation, we have utilized a dimolybdenum core with 4-methyl­benzoate and tetra­hydro­furan (THF) ligands to extend the electronic effect of varying substituents. Here we present the crystal structure and synthesis of *tetra­kis*(*μ*-4-methyl­benzoato-*κ* ^2^ *O:O^’^*)-bis­(tetra­hydro­furan) dimolyb­den­um(II) solvate [Mo_2_(*p*-O_2_CC_6_H_4_CH_3_)_4_·2(C_4_H_8_O)]·C_4_H_8_O. The presence of an electron-donating methyl group on the bridging benzoate ligands results in a minor elongation of the dimolybdenum bond with respect to the parent benzoate compound and compression in comparison to a benzoate complex with an electron-withdrawing tri­fluoro­methyl group. 
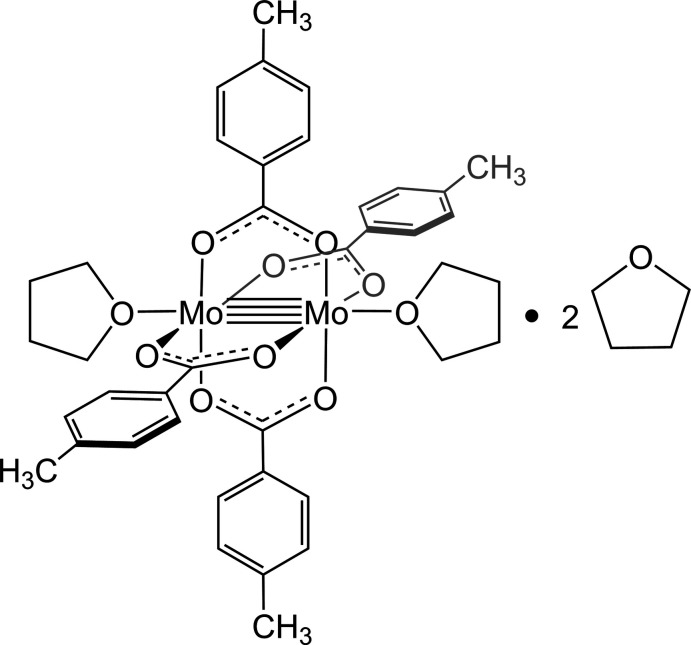


## 2. Structural commentary

**Figure 1:**
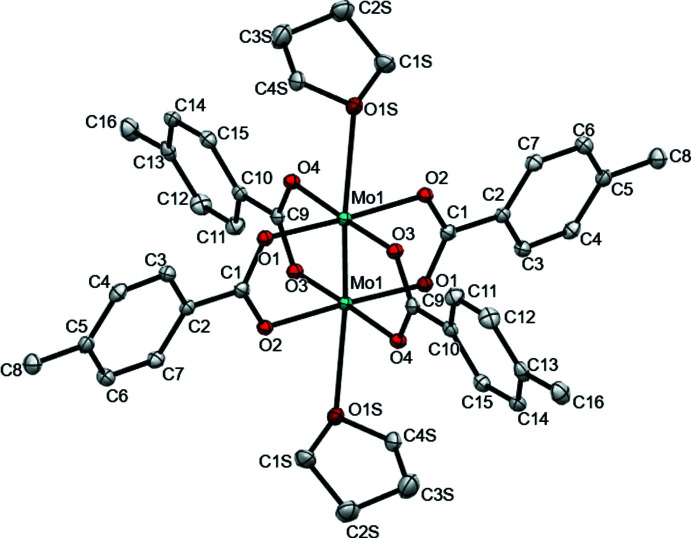
Ellipsoid plot of the title compound, with the atom-numbering scheme. Displacement ellipsoids are drawn at the 50% probability level. Hydrogen atoms and unbound THF solvent mol­ecules are omitted for clarity. Color scheme: C (gray), O (red), Mo (teal). The Mo1 atom connects to its symmetry-generated atom with an Mo1—Mo1^i^ bond length of 2.1012 (4) Å [symmetry code: (i) −*x* + 1, −*y*, −*z* + 1; Table 1 ▸]

The mol­ecular structure of the dimolybdenum complex, [Mo_2_(*p*-O_2_CC_6_H_4_CH_3_)_4_·2(C_4_H_8_O)] is presented in Fig. 1 ▸ as ascertained using single-crystal X-ray diffraction. The asymmetric unit contains half of the mol­ecule (Fig. 1 ▸), which upon inversion about the quadruple bond, yields the complete mol­ecular structure. Pertinent bond metrics for [Mo_2_(*p*-O_2_CC_6_H_4_CH_3_)_4_·2(C_4_H_8_O)]·2C_4_H_8_O were collected and compiled in Table 1 ▸. Complete tables of the structural metrics of the title compound are listed in the supporting information. The dimolybdenum bond distance is 2.1012 (4) Å, which is consistent with the previously reported Mo—Mo quadruple-bond distances of 2.06–2.17 Å (Cotton *et al.*, 2002 ▸). Noting that the dimolybdenum bond distance of the unsubstituted phenyl analogue, Mo_2_(O_2_CC_6_H_5_)_4_, is 2.096 (1) Å (Cotton *et al.*, 1978 ▸), the addition of the methyl group at the 4-position of the benzoate results in an increase of the dimolybdenum bond distance by 0.0053 (1) Å.

The electron-donating nature of the methyl-substituted benzoates is illuminated by comparing the p*K_a_* values of carboxyl­ate ligands [p*K*_a_ = 4.37 for *p*-O_2_CC_6_H_4_CH_3_, p*K*_a_ = 4.19 for O_2_CC_6_H_5_ (Hollingsworth *et al.*, 2002 ▸); p*K_a_* = 3.77 for *p*-O_2_CC_6_H_4_CF_3_ (Rumble, 2021 ▸)]. A comparative analysis of the dimolybdenum bond lengths of [Mo_2_(*p*-O_2_CC_6_H_4_CH_3_)_4_·(C_4_H_8_O)] and [Mo_2_(*p*-O_2_CC_6_H_4_CF_3_)·(C_4_H_8_O)] demonstrates that the addition of an electron-donating equatorial ligand does not lead to a distinguishable variation; the *d*(Mo—Mo) for [Mo_2_(*p*-O_2_CC_6_H_4_CF_3_)·(C_4_H_8_O)] is 2.1098 (7) Å (Aigeldinger *et al.*, 2022 ▸) and in this study *d*(Mo—Mo) for [Mo_2_(*p*-O_2_CC_6_H_4_CH_3_)·(C_4_H_8_O)] is 2.1012 (4) Å. Therefore, the addition of a ligand with electron-donating or withdrawing properties does not perturb the dimolybdenum quadruple-bond length. These observations support the findings of Han (2011 ▸) and Aigeldinger (Aigeldinger *et al.*, 2022 ▸), concluding that while holding the axial ligand (THF) constant, placing a series of *R* groups on the carboxyl­ate negligibly perturbs the Mo—Mo bond distance.

## 3. Supra­molecular features

**Figure 2:**
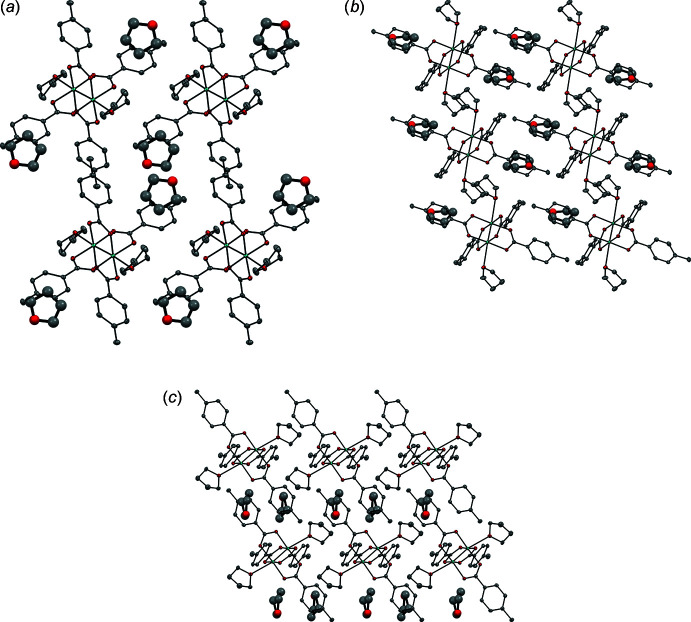
Crystal packing of the title compound shown along (*a*) the *a*-axis, (*b*) the *b*-axis, and (*c*) the *c*-axis. THF solvent mol­ecules are present in the lattice. Color scheme: C (gray), O (red), Mo (teal). Hydrogen atoms are omitted for clarity.

Mol­ecular packing arrangements are shown in Fig. 2 ▸. The structure was solved in the triclinic space group *P* 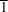 . Unbound THF mol­ecules are ordered in between *p*-O_2_CC_6_H_4_CH_3_ ligands of adjacent mol­ecules, along the *b*-axis, with the oxygen atom facing away from the metal center and toward the methyl groups.

The O2 oxygen atoms of the unbound THF solvent mol­ecules are located at distances of 4.178 (3) and 6.530 (4) Å from the C13 atoms of the *p*-O_2_CC_6_H_4_CH_3_ ligands of adjacent mol­ecules.

## 4. Database survey

A search in the Cambridge Structural Database (WebCSD, accessed November 2022; Groom *et al.*, 2016 ▸) for the CSD search fragment C_32_H_28_Mo_2_O_8_ for Mo_2_(*p*-O_2_CC_6_H_4_CH_3_)_4_ yielded no hits. The CSD search fragment C_40_H_44_Mo_2_O_10_ for [Mo_2_(*p*-O_2_CC_6_H_4_CH_3_)_4_·(C_4_H_8_O)] also yielded no hits. The CSD reference code for Mo_2_(O_2_CCH_3_)_4_ (Cotton *et al.*, 1974 ▸) is MOLACE01 and for Mo_2_(O_2_CC_6_H_5_)_4_ (Cotton *et al.*, 1978 ▸) is MOBZOA.

## 5. Synthesis and crystallization

**Figure 3:**
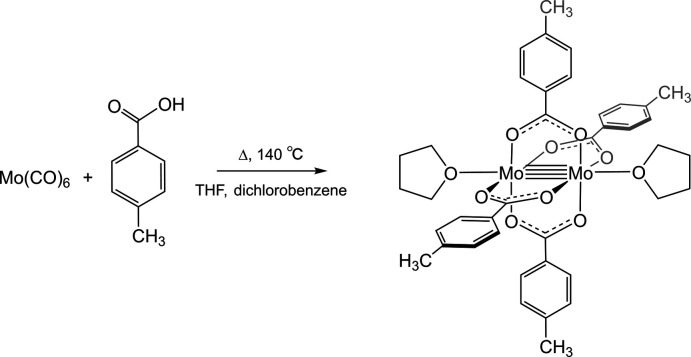
Synthesis scheme for [Mo_2_(*p*-O_2_CC_6_H_4_CH_3_)_4_(C_4_H_8_O)_2_].

Fig. 3 ▸ shows the overall synthetic reaction scheme. Molybdenum hexa­carbonyl [Mo(CO)_6_], *p*-toluic acid, anhydrous THF, and 1,2-di­chloro­benzene were purchased from Sigma-Aldrich. Mo(CO)_6_ and *p*-toluic acid were combined in an oven-dried flask with anhydrous THF and anhydrous 1,2-di­chloro­benzene. The reaction was heated under reflux for 48 h at 413 K under a dry N_2_ atmosphere (Pence *et al.*, 1999 ▸). The reaction mixture was cooled, dried, and washed with anhydrous di­chloro­methane and pentane.

The crystallization was prepared in a glove box. The crude product was dissolved in anhydrous THF, filtered, and recrystallized by vapor diffusion of pentane using a 6 by 50 mm borosilicate glass crystallization tube housed within a 20 mL glass vial. The assembly was allowed to stand at 238 K for 14 days. Orange block-shaped crystals were observed and harvested for X-ray diffraction analysis.

## 6. Refinement

Table 2 ▸ contains crystal data, data collection, and structure refinement details. A single orange block (0.220 mm × 0.180 mm × 0.140 mm) was chosen for single-crystal X-ray diffraction using a Bruker D8 goniometer equipped with an Photon100 CMOS detector. Data were collected as a series of φ and/or ω scans. Data integration down to 0.84 Å resolution was carried out using *SAINT* V8.37A with reflection spot size optimization. Absorption corrections were made with the program *SADABS2016/2* (Krause *et al.*, 2015 ▸). Space-group assignments were determined by examination of systematic absences, *E*-statistics, and successive refinement of the structures. The structure was solved by the intrinsic phasing method and refined by least-squares methods also using *SHELXT2014/5* and *SHELXL2014/7* with the *OLEX2* (Dolomanov *et al.*, 2009 ▸) inter­face. The program *PLATON* (Spek, 2020 ▸) was employed to confirm the absence of higher symmetry space groups. All non-H atoms, including the disorder fragment, were located in difference-Fourier maps, and then refined anisotropically. Outlier reflections were omitted from refinement when appropriate. Hydrogen atoms on C atoms were placed at idealized positions and refined using a riding model. The isotropic displacement parameters of all hydrogen atoms were fixed to 1.2 times the atoms they are linked to (1.5 times for methyl groups). Crystallographic refinement details, including the software employed, have been delineated within the crystallographic information (*.cif).

## Supplementary Material

Crystal structure: contains datablock(s) I. DOI: 10.1107/S2056989023001457/ex2066sup1.cif

Structure factors: contains datablock(s) I. DOI: 10.1107/S2056989023001457/ex2066Isup4.hkl

CCDC reference: 2242699

Additional supporting information:  crystallographic information; 3D view; checkCIF report

## Figures and Tables

**Table 1:** Selected geometric parameters (Å, °)

Mo1—O3	2.0955 (15)	Mo1—O2^i^	2.1119 (15)
Mo1—O1	2.1011 (15)	Mo1—O4^i^	2.1177 (15)
Mo1—Mo1^i^	2.1012 (4)	Mo1—O1*S*	2.5980 (16)
			
O3—Mo1—Mo1^i^	92.27 (4)	Mo1^i^—Mo1—O2^i^	90.28 (4)
O1—Mo1—Mo1^i^	93.14 (4)	Mo1^i^—Mo1—O4^i^	91.23 (4)

Symmetry code: (i) 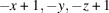 .

**Table 2:** Experimental details

Crystal data	
Chemical formula	[Mo(C_8_H_7_O_2_)_4_(C_4_H_8_O)_2_]·2C_4_H_8_O
*M*_r_	1020.84
Crystal system, space group	Triclinic, *P* 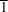
Temperature (K)	100
*a*, *b*, *c* (Å)	9.3923 (7), 10.6505 (8), 12.2955 (9)
α, β, γ (°)	78.001 (2), 74.374 (2), 69.853 (2)
*V* (Å^3^)	1102.96 (14)
*Z*	1
Radiation type	Mo *K*α
μ (mm^−1^)	0.63
Crystal size (mm)	0.22 × 0.18 × 0.14

Data collection	
Diffractometer	Bruker D8 goniometer with Photon 100 CMOS detector
Absorption correction	Multi-scan (*SADABS*; Krause *et al.*, 2015 ▸)
*T*_min_, *T*_max_	0.731, 0.767
No. of measured, independent and observed [*I* > 2σ(*I*)] reflections	22892, 3909, 3693
*R*_int_	0.027
(sin θ/λ)_max_ (Å^−1^)	0.597

Refinement	
*R*[*F* ^2^ > 2σ(*F* ^2^)], *wR*(*F* ^2^), *S*	0.026, 0.067, 1.09
No. of reflections	3909
No. of parameters	282
H-atom treatment	H-atom parameters constrained
Δρ_max_, Δρ_min_ (e Å^−3^)	0.90, −0.65

Computer programs: *SAINT* (Bruker, 2015 ▸), *SHELXT2014/5* (Sheldrick, 2015*a* ▸), *SHELXL2014/7* (Sheldrick, 2015*b* ▸) and *SHELXTL* (Sheldrick, 2008 ▸).

## supplementary crystallographic information

### Crystal data

**Table:**

[Mo_2_(C_8_H_7_O_2_)_4_(C_4_H_8_O)_2_]·2C_4_H_8_O	*Z* = 1
*M**_r_* = 1020.84	*F*(000) = 528
Triclinic, *P*1	*D*_x_ = 1.537 Mg m^−^^3^
*a* = 9.3923 (7) Å	Mo *K*α radiation, λ = 0.71073 Å
*b* = 10.6505 (8) Å	Cell parameters from 9495 reflections
*c* = 12.2955 (9) Å	θ = 2.4–27.2°
α = 78.001 (2)°	µ = 0.63 mm^−^^1^
β = 74.374 (2)°	*T* = 100 K
γ = 69.853 (2)°	Block, orange
*V* = 1102.96 (14) Å^3^	0.22 × 0.18 × 0.14 mm

### Data collection

**Table:**

Bruker D8 goniometer with Photon 100 CMOS detector diffractometer	3693 reflections with *I* > 2σ(*I*)
Radiation source: IµS microfocus tube	*R*_int_ = 0.027
ω and phi scans	θ_max_ = 25.1°, θ_min_ = 2.4°
Absorption correction: multi-scan (SADABS; Krause *et al.*, 2015)	*h* = −11→11
*T*_min_ = 0.731, *T*_max_ = 0.767	*k* = −12→12
22892 measured reflections	*l* = −14→14
3909 independent reflections	

### Refinement

**Table:**

Refinement on *F*^2^	Primary atom site location: dual
Least-squares matrix: full	Hydrogen site location: inferred from neighbouring sites
*R*[*F*^2^ > 2σ(*F*^2^)] = 0.026	H-atom parameters constrained
*wR*(*F*^2^) = 0.067	*w* = 1/[σ^2^(*F*_o_^2^) + (0.0302*P*)^2^ + 1.5476*P*] where *P* = (*F*_o_^2^ + 2*F*_c_^2^)/3
*S* = 1.09	(Δ/σ)_max_ = 0.002
3909 reflections	Δρ_max_ = 0.90 e Å^−^^3^
282 parameters	Δρ_min_ = −0.65 e Å^−^^3^
0 restraints	

### Special details

**Table:**

Geometry. All esds (except the esd in the dihedral angle between two l.s. planes) are estimated using the full covariance matrix. The cell esds are taken into account individually in the estimation of esds in distances, angles and torsion angles; correlations between esds in cell parameters are only used when they are defined by crystal symmetry. An approximate (isotropic) treatment of cell esds is used for estimating esds involving l.s. planes.
Refinement. Refinement of F^2^ against ALL reflections. The weighted R-factor wR and goodness of fit S are based on F^2^, conventional R-factors R are based on F, with F set to zero for negative F^2^. The threshold expression of F^2^ > 2sigma(F^2^) is used only for calculating R-factors(gt) etc. and is not relevant to the choice of reflections for refinement. R-factors based on F^2^ are statistically about twice as large as those based on F, and R- factors based on all data will be even larger. All non-H atoms were located in difference-Fourier maps, and then refined anisotropically.

### Fractional atomic coordinates and isotropic or equivalent isotropic displacement parameters (Å^2^)

**Table:**

	*x*	*y*	*z*	*U*_iso_*/*U*_eq_	
Mo1	0.39274 (2)	0.06805 (2)	0.53300 (2)	0.01099 (8)	
O1	0.46193 (18)	0.23761 (15)	0.44960 (13)	0.0131 (3)	
O2	0.68863 (17)	0.09443 (15)	0.37715 (13)	0.0127 (3)	
O3	0.46834 (18)	0.07766 (15)	0.67574 (13)	0.0132 (3)	
O4	0.69515 (18)	−0.07015 (15)	0.60914 (13)	0.0129 (3)	
C1	0.5992 (3)	0.2138 (2)	0.38833 (19)	0.0134 (5)	
C2	0.6581 (3)	0.3276 (2)	0.33066 (19)	0.0130 (5)	
C3	0.5784 (3)	0.4587 (2)	0.35819 (19)	0.0149 (5)	
H3	0.4818	0.4759	0.4117	0.018*	
C4	0.6392 (3)	0.5629 (2)	0.3081 (2)	0.0170 (5)	
H4	0.5854	0.6506	0.3294	0.020*	
C5	0.7784 (3)	0.5418 (2)	0.2267 (2)	0.0164 (5)	
C6	0.8555 (3)	0.4114 (2)	0.1982 (2)	0.0167 (5)	
H6	0.9496	0.3951	0.1421	0.020*	
C7	0.7978 (3)	0.3058 (2)	0.24985 (19)	0.0152 (5)	
H7	0.8535	0.2175	0.2303	0.018*	
C8	0.8424 (3)	0.6562 (2)	0.1713 (2)	0.0211 (5)	
H8A	0.7822	0.7132	0.1154	0.032*	
H8B	0.9513	0.6202	0.1329	0.032*	
H8C	0.8357	0.7098	0.2294	0.032*	
C9	0.6052 (3)	0.0033 (2)	0.68511 (19)	0.0134 (5)	
C10	0.6588 (3)	0.0043 (2)	0.78726 (19)	0.0135 (5)	
C11	0.5778 (3)	0.1055 (2)	0.8573 (2)	0.0187 (5)	
H11	0.4876	0.1734	0.8393	0.022*	
C12	0.6274 (3)	0.1077 (2)	0.9520 (2)	0.0201 (5)	
H12	0.5719	0.1784	0.9976	0.024*	
C13	0.7570 (3)	0.0086 (2)	0.9827 (2)	0.0170 (5)	
C14	0.8374 (3)	−0.0929 (2)	0.9130 (2)	0.0160 (5)	
H14	0.9261	−0.1620	0.9323	0.019*	
C15	0.7901 (3)	−0.0945 (2)	0.81614 (19)	0.0154 (5)	
H15	0.8475	−0.1635	0.7690	0.018*	
C16	0.8066 (3)	0.0121 (3)	1.0881 (2)	0.0225 (5)	
H16A	0.7314	−0.0101	1.1555	0.034*	
H16B	0.8114	0.1025	1.0882	0.034*	
H16C	0.9093	−0.0538	1.0892	0.034*	
O1S	0.10283 (19)	0.19386 (16)	0.61146 (14)	0.0175 (4)	
C1S	−0.0151 (3)	0.1276 (3)	0.6333 (2)	0.0256 (6)	
H1SA	−0.0575	0.1121	0.7161	0.031*	
H1SB	0.0293	0.0395	0.6034	0.031*	
C2S	−0.1417 (3)	0.2203 (3)	0.5732 (2)	0.0280 (6)	
H2SA	−0.1805	0.1685	0.5360	0.034*	
H2SB	−0.2297	0.2731	0.6273	0.034*	
C3S	−0.0621 (3)	0.3109 (3)	0.4864 (3)	0.0319 (7)	
H3SA	−0.0080	0.2693	0.4155	0.038*	
H3SB	−0.1370	0.3997	0.4679	0.038*	
C4S	0.0514 (3)	0.3239 (2)	0.5461 (2)	0.0207 (5)	
H4SA	0.1401	0.3459	0.4902	0.025*	
H4SB	0.0005	0.3954	0.5966	0.025*	
O2S	0.2645 (3)	0.5866 (2)	0.05605 (18)	0.0418 (5)	
C5S	0.2502 (4)	0.5990 (3)	0.1730 (3)	0.0358 (7)	
H5SA	0.1489	0.6638	0.2027	0.043*	
H5SB	0.3343	0.6304	0.1809	0.043*	
C6S	0.2628 (3)	0.4590 (3)	0.2366 (2)	0.0319 (6)	
H6SA	0.3031	0.4452	0.3061	0.038*	
H6SB	0.1613	0.4413	0.2575	0.038*	
C7S	0.3761 (4)	0.3726 (3)	0.1498 (3)	0.0346 (7)	
H7SA	0.3680	0.2800	0.1655	0.042*	
H7SB	0.4842	0.3684	0.1463	0.042*	
C8S	0.3238 (4)	0.4464 (3)	0.0429 (3)	0.0358 (7)	
H8SA	0.4121	0.4303	−0.0236	0.043*	
H8SB	0.2420	0.4152	0.0310	0.043*	

### Atomic displacement parameters (Å^2^)

**Table:**

	*U*^11^	*U*^22^	*U*^33^	*U*^12^	*U*^13^	*U*^23^
Mo1	0.01006 (11)	0.01080 (11)	0.01143 (11)	−0.00298 (7)	−0.00185 (7)	−0.00096 (7)
O1	0.0120 (8)	0.0122 (8)	0.0138 (8)	−0.0031 (6)	−0.0019 (6)	−0.0013 (6)
O2	0.0110 (8)	0.0115 (8)	0.0140 (8)	−0.0029 (6)	−0.0010 (6)	−0.0016 (6)
O3	0.0117 (8)	0.0131 (8)	0.0141 (8)	−0.0027 (6)	−0.0024 (6)	−0.0022 (6)
O4	0.0117 (8)	0.0137 (8)	0.0127 (8)	−0.0035 (6)	−0.0016 (6)	−0.0025 (6)
C1	0.0132 (12)	0.0167 (12)	0.0114 (11)	−0.0046 (9)	−0.0043 (9)	−0.0018 (9)
C2	0.0130 (11)	0.0145 (11)	0.0120 (11)	−0.0043 (9)	−0.0045 (9)	0.0001 (9)
C3	0.0124 (11)	0.0167 (12)	0.0142 (11)	−0.0036 (9)	−0.0022 (9)	−0.0012 (9)
C4	0.0190 (12)	0.0113 (11)	0.0201 (12)	−0.0019 (9)	−0.0071 (10)	−0.0013 (9)
C5	0.0169 (12)	0.0169 (12)	0.0164 (12)	−0.0061 (10)	−0.0075 (10)	0.0027 (9)
C6	0.0147 (12)	0.0184 (12)	0.0162 (12)	−0.0061 (10)	−0.0019 (9)	−0.0011 (9)
C7	0.0155 (12)	0.0136 (11)	0.0158 (12)	−0.0029 (9)	−0.0034 (9)	−0.0028 (9)
C8	0.0208 (13)	0.0165 (12)	0.0249 (13)	−0.0073 (10)	−0.0036 (10)	0.0010 (10)
C9	0.0138 (12)	0.0102 (11)	0.0155 (12)	−0.0053 (9)	−0.0022 (9)	0.0015 (9)
C10	0.0131 (11)	0.0142 (11)	0.0136 (11)	−0.0072 (9)	−0.0018 (9)	0.0009 (9)
C11	0.0187 (12)	0.0156 (12)	0.0200 (13)	−0.0032 (10)	−0.0041 (10)	−0.0022 (10)
C12	0.0233 (13)	0.0172 (12)	0.0190 (12)	−0.0050 (10)	−0.0012 (10)	−0.0073 (10)
C13	0.0187 (12)	0.0210 (12)	0.0146 (12)	−0.0130 (10)	−0.0012 (9)	−0.0003 (9)
C14	0.0124 (11)	0.0194 (12)	0.0159 (12)	−0.0068 (10)	−0.0028 (9)	0.0014 (9)
C15	0.0143 (12)	0.0160 (12)	0.0150 (12)	−0.0056 (9)	0.0001 (9)	−0.0030 (9)
C16	0.0254 (14)	0.0297 (14)	0.0159 (12)	−0.0129 (11)	−0.0040 (10)	−0.0031 (10)
O1S	0.0154 (8)	0.0144 (8)	0.0207 (9)	−0.0039 (7)	−0.0050 (7)	0.0020 (7)
C1S	0.0168 (13)	0.0222 (13)	0.0380 (16)	−0.0108 (11)	−0.0065 (11)	0.0047 (11)
C2S	0.0230 (14)	0.0279 (15)	0.0367 (16)	−0.0105 (12)	−0.0138 (12)	0.0023 (12)
C3S	0.0328 (16)	0.0319 (16)	0.0329 (16)	−0.0119 (13)	−0.0164 (13)	0.0076 (12)
C4S	0.0186 (13)	0.0149 (12)	0.0242 (13)	−0.0051 (10)	−0.0027 (10)	0.0041 (10)
O2S	0.0511 (14)	0.0390 (12)	0.0312 (11)	−0.0086 (11)	−0.0081 (10)	−0.0058 (9)
C5S	0.0371 (17)	0.0394 (17)	0.0315 (16)	−0.0117 (14)	−0.0052 (13)	−0.0086 (13)
C6S	0.0288 (15)	0.0413 (17)	0.0281 (15)	−0.0157 (13)	−0.0076 (12)	0.0005 (13)
C7S	0.0352 (17)	0.0314 (16)	0.0390 (17)	−0.0140 (13)	−0.0055 (13)	−0.0046 (13)
C8S	0.0331 (16)	0.0350 (17)	0.0391 (17)	−0.0134 (13)	−0.0022 (13)	−0.0065 (13)

### Geometric parameters (Å, º)

**Table:**

Mo1—O3	2.0955 (15)	C13—C14	1.396 (3)
Mo1—O1	2.1011 (15)	C13—C16	1.501 (3)
Mo1—Mo1^i^	2.1012 (4)	C14—C15	1.384 (3)
Mo1—O2^i^	2.1119 (15)	C14—H14	0.9500
Mo1—O4^i^	2.1177 (15)	C15—H15	0.9500
Mo1—O1S	2.5980 (16)	C16—H16A	0.9800
O1—C1	1.275 (3)	C16—H16B	0.9800
O2—C1	1.271 (3)	C16—H16C	0.9800
O2—Mo1^i^	2.1119 (15)	O1S—C4S	1.443 (3)
O3—C9	1.278 (3)	O1S—C1S	1.449 (3)
O4—C9	1.274 (3)	C1S—C2S	1.515 (4)
O4—Mo1^i^	2.1177 (15)	C1S—H1SA	0.9900
C1—C2	1.473 (3)	C1S—H1SB	0.9900
C2—C7	1.396 (3)	C2S—C3S	1.506 (4)
C2—C3	1.402 (3)	C2S—H2SA	0.9900
C3—C4	1.378 (3)	C2S—H2SB	0.9900
C3—H3	0.9500	C3S—C4S	1.503 (4)
C4—C5	1.395 (3)	C3S—H3SA	0.9900
C4—H4	0.9500	C3S—H3SB	0.9900
C5—C6	1.397 (3)	C4S—H4SA	0.9900
C5—C8	1.499 (3)	C4S—H4SB	0.9900
C6—C7	1.378 (3)	O2S—C8S	1.430 (4)
C6—H6	0.9500	O2S—C5S	1.438 (4)
C7—H7	0.9500	C5S—C6S	1.514 (4)
C8—H8A	0.9800	C5S—H5SA	0.9900
C8—H8B	0.9800	C5S—H5SB	0.9900
C8—H8C	0.9800	C6S—C7S	1.497 (4)
C9—C10	1.477 (3)	C6S—H6SA	0.9900
C10—C15	1.393 (3)	C6S—H6SB	0.9900
C10—C11	1.397 (3)	C7S—C8S	1.497 (4)
C11—C12	1.373 (4)	C7S—H7SA	0.9900
C11—H11	0.9500	C7S—H7SB	0.9900
C12—C13	1.392 (4)	C8S—H8SA	0.9900
C12—H12	0.9500	C8S—H8SB	0.9900
			
O3—Mo1—O1	89.23 (6)	C13—C14—H14	119.5
O3—Mo1—Mo1^i^	92.27 (4)	C14—C15—C10	120.5 (2)
O1—Mo1—Mo1^i^	93.14 (4)	C14—C15—H15	119.8
O3—Mo1—O2^i^	89.85 (6)	C10—C15—H15	119.8
O1—Mo1—O2^i^	176.49 (6)	C13—C16—H16A	109.5
Mo1^i^—Mo1—O2^i^	90.28 (4)	C13—C16—H16B	109.5
O3—Mo1—O4^i^	176.30 (6)	H16A—C16—H16B	109.5
O1—Mo1—O4^i^	89.37 (6)	C13—C16—H16C	109.5
Mo1^i^—Mo1—O4^i^	91.23 (4)	H16A—C16—H16C	109.5
O2^i^—Mo1—O4^i^	91.34 (6)	H16B—C16—H16C	109.5
O3—Mo1—O1S	94.89 (6)	C4S—O1S—C1S	108.66 (18)
O1—Mo1—O1S	97.74 (6)	C4S—O1S—Mo1	111.78 (13)
Mo1^i^—Mo1—O1S	167.04 (4)	C1S—O1S—Mo1	120.84 (14)
O2^i^—Mo1—O1S	78.97 (5)	O1S—C1S—C2S	106.7 (2)
O4^i^—Mo1—O1S	81.90 (6)	O1S—C1S—H1SA	110.4
C1—O1—Mo1	116.16 (14)	C2S—C1S—H1SA	110.4
C1—O2—Mo1^i^	118.39 (14)	O1S—C1S—H1SB	110.4
C9—O3—Mo1	117.32 (14)	C2S—C1S—H1SB	110.4
C9—O4—Mo1^i^	117.29 (14)	H1SA—C1S—H1SB	108.6
O2—C1—O1	122.0 (2)	C3S—C2S—C1S	103.6 (2)
O2—C1—C2	118.7 (2)	C3S—C2S—H2SA	111.0
O1—C1—C2	119.3 (2)	C1S—C2S—H2SA	111.0
C7—C2—C3	118.6 (2)	C3S—C2S—H2SB	111.0
C7—C2—C1	120.3 (2)	C1S—C2S—H2SB	111.0
C3—C2—C1	121.0 (2)	H2SA—C2S—H2SB	109.0
C4—C3—C2	120.4 (2)	C4S—C3S—C2S	102.6 (2)
C4—C3—H3	119.8	C4S—C3S—H3SA	111.2
C2—C3—H3	119.8	C2S—C3S—H3SA	111.2
C3—C4—C5	121.3 (2)	C4S—C3S—H3SB	111.2
C3—C4—H4	119.4	C2S—C3S—H3SB	111.2
C5—C4—H4	119.4	H3SA—C3S—H3SB	109.2
C4—C5—C6	117.9 (2)	O1S—C4S—C3S	105.1 (2)
C4—C5—C8	120.9 (2)	O1S—C4S—H4SA	110.7
C6—C5—C8	121.2 (2)	C3S—C4S—H4SA	110.7
C7—C6—C5	121.3 (2)	O1S—C4S—H4SB	110.7
C7—C6—H6	119.3	C3S—C4S—H4SB	110.7
C5—C6—H6	119.3	H4SA—C4S—H4SB	108.8
C6—C7—C2	120.4 (2)	C8S—O2S—C5S	108.1 (2)
C6—C7—H7	119.8	O2S—C5S—C6S	105.4 (2)
C2—C7—H7	119.8	O2S—C5S—H5SA	110.7
C5—C8—H8A	109.5	C6S—C5S—H5SA	110.7
C5—C8—H8B	109.5	O2S—C5S—H5SB	110.7
H8A—C8—H8B	109.5	C6S—C5S—H5SB	110.7
C5—C8—H8C	109.5	H5SA—C5S—H5SB	108.8
H8A—C8—H8C	109.5	C7S—C6S—C5S	101.5 (2)
H8B—C8—H8C	109.5	C7S—C6S—H6SA	111.5
O4—C9—O3	121.8 (2)	C5S—C6S—H6SA	111.5
O4—C9—C10	119.8 (2)	C7S—C6S—H6SB	111.5
O3—C9—C10	118.3 (2)	C5S—C6S—H6SB	111.5
C15—C10—C11	118.5 (2)	H6SA—C6S—H6SB	109.3
C15—C10—C9	121.3 (2)	C6S—C7S—C8S	101.3 (2)
C11—C10—C9	120.1 (2)	C6S—C7S—H7SA	111.5
C12—C11—C10	120.5 (2)	C8S—C7S—H7SA	111.5
C12—C11—H11	119.7	C6S—C7S—H7SB	111.5
C10—C11—H11	119.7	C8S—C7S—H7SB	111.5
C11—C12—C13	121.5 (2)	H7SA—C7S—H7SB	109.3
C11—C12—H12	119.2	O2S—C8S—C7S	107.0 (2)
C13—C12—H12	119.2	O2S—C8S—H8SA	110.3
C12—C13—C14	117.9 (2)	C7S—C8S—H8SA	110.3
C12—C13—C16	120.3 (2)	O2S—C8S—H8SB	110.3
C14—C13—C16	121.8 (2)	C7S—C8S—H8SB	110.3
C15—C14—C13	121.0 (2)	H8SA—C8S—H8SB	108.6
C15—C14—H14	119.5		
			
Mo1^i^—O2—C1—O1	−1.7 (3)	O4—C9—C10—C11	165.2 (2)
Mo1^i^—O2—C1—C2	176.87 (14)	O3—C9—C10—C11	−14.6 (3)
Mo1—O1—C1—O2	0.8 (3)	C15—C10—C11—C12	0.4 (4)
Mo1—O1—C1—C2	−177.72 (15)	C9—C10—C11—C12	−179.6 (2)
O2—C1—C2—C7	11.2 (3)	C10—C11—C12—C13	−1.3 (4)
O1—C1—C2—C7	−170.2 (2)	C11—C12—C13—C14	0.9 (4)
O2—C1—C2—C3	−166.7 (2)	C11—C12—C13—C16	−178.8 (2)
O1—C1—C2—C3	11.9 (3)	C12—C13—C14—C15	0.3 (3)
C7—C2—C3—C4	−1.5 (3)	C16—C13—C14—C15	−180.0 (2)
C1—C2—C3—C4	176.4 (2)	C13—C14—C15—C10	−1.2 (3)
C2—C3—C4—C5	1.9 (4)	C11—C10—C15—C14	0.8 (3)
C3—C4—C5—C6	−0.7 (3)	C9—C10—C15—C14	−179.2 (2)
C3—C4—C5—C8	179.0 (2)	C4S—O1S—C1S—C2S	2.6 (3)
C4—C5—C6—C7	−1.0 (3)	Mo1—O1S—C1S—C2S	−128.57 (18)
C8—C5—C6—C7	179.3 (2)	O1S—C1S—C2S—C3S	19.8 (3)
C5—C6—C7—C2	1.5 (4)	C1S—C2S—C3S—C4S	−33.5 (3)
C3—C2—C7—C6	−0.2 (3)	C1S—O1S—C4S—C3S	−24.1 (3)
C1—C2—C7—C6	−178.1 (2)	Mo1—O1S—C4S—C3S	111.77 (19)
Mo1^i^—O4—C9—O3	−1.3 (3)	C2S—C3S—C4S—O1S	35.7 (3)
Mo1^i^—O4—C9—C10	178.91 (15)	C8S—O2S—C5S—C6S	−14.8 (3)
Mo1—O3—C9—O4	2.6 (3)	O2S—C5S—C6S—C7S	34.5 (3)
Mo1—O3—C9—C10	−177.62 (14)	C5S—C6S—C7S—C8S	−39.8 (3)
O4—C9—C10—C15	−14.9 (3)	C5S—O2S—C8S—C7S	−11.0 (3)
O3—C9—C10—C15	165.3 (2)	C6S—C7S—C8S—O2S	32.4 (3)

Symmetry code: (i) −*x*+1, −*y*, −*z*+1.
